# Rigorous Policy-Making Amid COVID-19 and Beyond: Literature Review and Critical Insights

**DOI:** 10.3390/ijerph182312447

**Published:** 2021-11-26

**Authors:** Zhaohui Su

**Affiliations:** Center on Smart and Connected Health Technologies, Mays Cancer Center, School of Nursing, UT Health San Antonio, San Antonio, TX 78229, USA; szh@utexas.edu

**Keywords:** COVID-19, health policy, public health, PADS, people-centered

## Abstract

Policies shape society. Public health policies are of particular importance, as they often dictate matters in life and death. Accumulating evidence indicates that good-intentioned COVID-19 policies, such as shelter-in-place measures, can often result in unintended consequences among vulnerable populations such as nursing home residents and domestic violence victims. Thus, to shed light on the issue, this study aimed to identify policy-making processes that have the potential of developing policies that could induce optimal desirable outcomes with limited to no unintended consequences amid the pandemic and beyond. **Methods:** A literature review was conducted in PubMed, PsycINFO, and Scopus to answer the research question. To better structure the review and the subsequent analysis, theoretical frameworks such as the social ecological model were adopted to guide the process. **Results:** The findings suggested that: (1) people-centered; (2) artificial intelligence (AI)-powered; (3) data-driven, and (4) supervision-enhanced policy-making processes could help society develop policies that have the potential to yield desirable outcomes with limited unintended consequences. To leverage these strategies’ interconnectedness, the people-centered, AI-powered, data-driven, and supervision-enhanced (PADS) model of policy making was subsequently developed. **Conclusions:** The PADS model can develop policies that have the potential to induce optimal outcomes and limit or eliminate unintended consequences amid COVID-19 and beyond. Rather than serving as a definitive answer to problematic COVID-19 policy-making practices, the PADS model could be best understood as one of many promising frameworks that could bring the pandemic policy-making process more in line with the interests of societies at large; in other words, more cost-effectively, and consistently anti-COVID and pro-human.

## 1. Background

As much as policies shape society, they create it as well [1]. The change can be either slow or fast—depending on the context, newly found commonalities, communities, cultures, if not new reckonings among the civilizations, can either occur incrementally or with lightning speed [2,3,4]. Take COVID-19 prevention policies, for instance. Ranging from loose measures to long-term mandates, COVID-19 policies have created communities (e.g., mask supporters, anti-vaxxers, conspiracy theorists, and citizen vigilantes) [5,6,7], cultures (e.g., the xenophobic culture, the civic culture) [8,9,10], and perhaps most importantly, new understandings of the shared vulnerabilities and strengthens of the civilization (e.g., the peril of extremely tiny viruses, the power of small vials of vaccines, and the promise of victory-minded humanity) [11,12,13].

Public policies can be understood as the “purposive course of action followed by an actor or a set of actors in dealing with a problem or matter of concern” [14], which are often “formal, legally-binding measures adopted by legislative and administrative units of government” [15]. Overall, public policies are arbitrary rules and regulations developed to create social goods [16]. Ranging from shelter-in-place measures to lockdown mandates, and masking rules to vaccine regulations, one common denominator of these policies is their ability to curb the spread of the pandemic, and in turn, COVID-19 infections, hospitalizations, and deaths [17,18,19]. In an epidemiological modeling study across eight countries, researchers found that an additional delay of imposing lockdown measures amid COVID-19 outbreaks for one week could result in half a million deaths that could have been avoided [20]. In a similar vein, an early implementation of stringent public policies on physical distancing and an early lifting of these policies are the main reasons the state of California had successfully controlled the COVID-19 outbreak first [21], and only later became the first state in the U.S. that surpassed 500,000 confirmed cases and 10,000 deaths [22,23].

However, it is important to note that public policies could also result in unintended consequences. A growing body of research indicates that separating people from their familiar routines and social environments could have devastating effects on their physical and psychological health [24,25,26]. Furthermore, evidence indicates that COVID-19 physical distancing measures could cause mental disorders including distress, anxiety, depression, and suicidal behaviors [27,28,29]. This might be especially true among vulnerable populations—older adults, domestic violence victims, racial/sexual minorities, and other underserved communities were among those who have been shouldering the most pronounced adverse impacts across the pandemic [30,31,32,33].

Take nursing home residents, for instance. A key characteristic of nursing home residents is that they have either lost or are losing their abilities to take care of themselves, a situation that is particularly pronounced among those who suffer from cognitive impairments such as dementia [31]. Amid COVID-19, many nursing home residents were found to have been left for days without access to care, food, or water, let alone basic medicines, and many of them died during the abandonment [34]. While nursing homes are often plagued with various issues [35,36,37], elder abuse and neglect have rarely been this glaring prior to the pandemic [38,39]. One way to address these unintended consequences is via addressing their root cause—rather than scrambling to construct piecemeal policies at the eleventh hour, rigorously and pre-emptively developing policies, such as via evidence-based policy-making processes, may hold the key [40].

Evidence-based policy making can be understood as the law-making process that is guided by and developed on the basis of evidence [41]. A rich body of evidence suggests that evidence-based policy making can provide considerable benefits to society at large [42]. However, it is important to note that evidence-based policy making is not without flaws [43,44,45,46], many of which have either been highlighted or magnified amid the pandemic [47,48]. Conventional policy making often follows a range of one-directional steps, including agenda setting, policy formulation, policy adoption and application, and policy evaluation [49]. This means that in order for the resultant policies to be evidence-based, reflective of people’s needs, and have the potential to yield positive outcomes, the policy-making process is often thoroughly planned, detail-rich, time-consuming, and resource-dependent [50]—parameters that most of the pandemic-era policy-making might not be able to meet.

In other words, the unprecedented nature of the pandemic has effectively deprived policy makers of the time and planning needed to develop most conventional policies pre-emptively, let alone evidence-based ones that might be even more resource-demanding. Second, the fast-evolving characteristics of the pandemic led to the inevitability that, most, if not all, policies developed based on the conventional stage-oriented policy-making procedures would significantly lag reality. As seen amid the pandemic, “facts” and “truisms”, such as “evidence-based” predictions that claim that summer 2021 is when the pandemic would end, might sound naïve, if not juvenile, in light of the Delta-disturbed reality [51]. This means that policies that are developed on old evidence, even if it is one month old, may offer little to no utility to society at large. Third, due to a lack of clear understanding of and consensus on what could be classified as “evidence” [52], as seen amid COVID-19, oftentimes even anecdotal stories and personal opinions, if not gut feelings, have been enlisted as the “evidence” upon which policy makers alike based their pandemic policies [53].

These drawbacks, in turn, could significantly compromise public health policies’ abilities to produce much-needed positive effects on society with limited to no unintended consequences. In other words, the conventional evidence-based policy-making processes may not be able to develop policies amid COVID-19 that could:

(1) yield desirable outcomes;
(2) produce little to no unintended consequences in light of the unique challenges of the pandemic. However, there is a dearth of insights available in the literature that could address the above-mentioned issues. Thus, to bridge the research gap, this study aimed to identify policy-making processes that have the potential to develop policies that could induce optimal desirable outcomes with limited to no unintended consequences amid the pandemic and beyond.

## 2. Methods

A literature review was conducted in PubMed, PsycINFO, and Scopus to identify rigorous policy-making processes that could develop competent policies with the potential of producing desirable outcomes and curbing unintended consequences amid the unique challenges of the COVID-19 pandemic. Overall, the research question raised in the study had three interconnected components: rigorous policy-making processes that could (1) produce desirable pandemic prevention outcomes, with (2) limited to no unintended consequences, in light of the (3) unique challenges of COVID-19. In this study, desirable pandemic prevention outcomes can be understood as reduced COVID-19 infections, hospitalizations, and deaths. Whereas “adverse unintended consequences” and “unintended consequences” are used interchangeably, referring to negative policy outcomes that were different from expected results.

The search was developed based on two overarching concepts: COVID-19 and policy making. An example PubMed search term can be found in Table 1. All records reviewed were published in English. To effectively address this three-pronged research aim, the review strategy was developed based on three themes:

(1) unique characteristics of COVID-19;
(2) rigorous policy-making processes;
(3) intended and unintended policy outcomes.

A set of eligibility criteria was adopted to screen the papers. Overall, articles were excluded if they:

(1) did not focus on COVID-19;
(2) did not center on the pandemic policy-making process;
(3) did not provide insights into approaches that could either improve intended outcomes or avoid unintended consequences.

To ensure up-to-date insights were included in the analysis, validated news reports were also reviewed. Furthermore, Google Scholar alerts were set up so that relevant and most updated insights could be reviewed and analyzed to further shed light on the research question. The initial search was first conducted on 8 August 2021, with the subsequent one conducted on 15 October 2021, to include updated insights in the review.

## 3. Theoretical Underpinning

To better guide the review process and the subsequent analysis, theoretical insights from behavioral sciences were adopted as the guiding framework. Specifically, the theoretical underpinning of the study was grounded in the extensively documented understanding that behaviors could be both rational and irrational, as seen in the well-debated strengths and weaknesses of value-expectancy theories such as the Theory of Planned Behavior [54,55,56], for instance. In other words, the study investigated the research question via an empirically based understanding that, regardless of the scale and scope of the impacts of the actions, the policy-making process can be both rational and irrational. Furthermore, drawing insights from the Social Ecological Model [57], which posits that social behaviors are often shaped by a multitude of factors with divergent strengthens of influences that often manifest on varied levels of society, the study adopted a solution-focused mindset to address the research question—with difficulties galore, what can be done to improve the efficacy of pandemic policy making with substantially limited or eliminated unintended consequences?

## 4. Results

In terms of peer-reviewed research, a total of 28 papers were included in the final review (see Table 2). The findings of the review were organized in accordance with the research aim—identify rigorous policy-making processes that could produce positive outcomes with limited to no unintended consequences in light of the unique challenges and opportunities of the COVID-19 pandemic. It is important to underscore that only a limited number of studies have investigated COVID-19 policies from a procedural perspective (e.g., [58,59,60]). In other words, instead of examining COVID-19 policies from a connected and comprehensive perspective, most of the research has focused on nuanced aspects of COVID-19 policy making, ranging from concrete facilitators (e.g., more effective prediction or monitoring of virus spread) and tangible barriers (e.g., lack of quality data), to the promises of advanced technology-enabled decision aids (e.g., AI-based decision models) (e.g., [61,62,63,64]) that could either hinder the smoothness or success of the policy-making process. However, while these insights could not answer the research question directly, they nonetheless were important and could be useful to tackle the research aim.

Therefore, in light of the novelty of the research question and the dearth of research insights available in the literature, all relevant insights were thoroughly reviewed and analyzed. Overall, based on the literature review and the subsequent analysis, the result suggests that policy-making processes incorporating the following strategies could develop policies that have the potential of yielding desirable outcomes with limited unintended consequences:

(1) people-centered: put people’s needs and wants at the center of the policy-making process, effectively prioritizing people over profits, politics, and the like [58,76,88,89,90,91,92,93];
(2) artificial intelligence (AI)-powered: incorporating intelligent and automatic decision-making mechanisms to ensure the policies are developed based on the most updated evidence [83,94,95,96,97,98];
(3) data-driven: the need to anchor key policy-making decisions with the support of empirical insights from quality data of optimal quantity and diversity [61,62,63,64,99,100,101,102];
(4) supervision-enhanced: oversight mechanisms that scrutinize the behaviors of both the policy makers and the AI systems to further enhance policies’ abilities to produce positive outcomes without incurring unintended consequences [69,103,104,105,106,107,108].

To leverage these strategies’ interconnectedness, the people-centered, AI-powered, data-driven, and supervision-enhanced (PADS) model of policy making was subsequently developed. In the following section, the PADS model will be discussed in detail.

## 5. Discussion

This study aims to identify policy-making processes that have the potential to develop policies that could induce optimal desirable outcomes with limited to no unintended consequences amid COVID-19 and beyond. This is one of the first studies that investigated solutions that could shed light on the bevy of policy-making issues the COVID-19 pandemic has introduced or intensified, ranging from opaque and questionable policy-making processes and unquestioned and unchecked power of policymakers, to the unprecedented pace seen in the erosion of health equity and implosion of public dissent partially caused by unintended consequences of COVID-19 policies [109,110,111]. Aiming to address key issues in current policy-making practices—poor adoption of rigorous data analytics, lack of accountability, and oversized dependence on individual decision makers or policy makers, the study identified strategies that could establish and sustain the rigor in COVID-19 policy-making processes—the people-centered, AI-powered, data-driven, and supervision-enhanced (PADS) model of policy-making.

### 5.1. People-Centered

People-centered means to put people’s needs and wants at the center of the policy-making process, effectively prioritizing people over profits, politics, and the like [58,76,88,89,90,91,92,93]. It is important to note that “people” refers to all key stakeholders that are involved in the policy-making process, ranging from decision makers such as policy makers, decision supervisors such as independent experts, and decision benefactors such as the general public. Overall, it is important to underscore that the degree to which people agree with policies is a critical factor in shaping COVID-19 containment outcomes [112,113]. As the literature shows, how individuals adopt and comply with public policies, whether due to belief in science [114], economic concerns [115], political ideology [116], or perceived people-friendliness of the public policies (e.g., duration of the lockdown) [117], may influence the effectiveness of these policies in controlling the spread of COVID-19. In other words, public health policies, such as lockdowns, self-isolation, and spatial distancing measures are only effective if the public acts willingly in accordance with these measures [112,113,114,115,116,117].

By prioritizing the people’s collective interests over individual profits, partisan politics, or the dominant powers at the moment, the people-centeredness of the policy-making process or the PADS model could not only safeguard personal and public health, but also prompt better adherence to the resultant COVID-19 policies. Take China’s zero-COVID policy, for instance. The zero-COVID policy is a unique disease elimination/eradiation policy that has two pillars:

(1) a “zero-tolerance” mindset that treats even single-digit positive COVID-19 cases or small disease outbreaks with the utmost urgency;
(2) a “zero-delay” action plan that employs and deploys robust and rigorous collective and corroborative actions and measures to subdue positive cases and squash potential outbreaks.

Understandably, the zero-COVID policy and its use of mass quarantines and lockdowns are often considered draconian [118], particularly in light of the ever-loosening pandemic measures adopted by other societies [119,120]. However, as the policy is people-centered—developed factoring in the needs of all members of the society, including vulnerable communities such as older adults, frontline workers, and volunteers [67,121,122], and possibly future short-term residents such as participants of the Beijing 2022 Winter Olympic Games [123]—the zero-COVID policy remains well supported and rigorously followed by the public [93].

### 5.2. AI-Powered

AI can be understood as machine programs or algorithms that are “able to mimic human intelligence” [124]. The AI-powered component of the PADS model emphasizes the importance of incorporating intelligent and automatic decision-making mechanisms to ensure the policies are developed based on the most updated and comprehensive evidence robustly analyzed [83,94,95,96,97,98]. Advanced AI systems can help policymakers to make more informed policies that are both reactive (retrospectively analyzing data to develop intelligent solutions) and proactive (predictive decision-making insights based on advanced modelling) in nature [125,126,127]. Furthermore, AI systems can often serve as the essential platform that enables other advanced technologies, ranging from augmented reality and virtual reality to mixed reality, if not the metaverse. In addition to AI’s role as the enabler, it can also perform the function of enhancer—improving performance of everyday services or commonplace information and communication technologies [125,126,127].

For example, AI-based systems could help government and health officers develop algorithms that incorporate in-depth and comprehensive insights gained on big data analysis on diverse data in the policy-making process, ranging from search queries, medical records, public health records, social media posts, online purchases, and wastewater to surveillance footage [83,124]. The potential of AI systems can be further amplified when coupled with 5G or 6G technologies; 6G, the sixth-generation networking technologies, can be understood as the next-generation transmission technique following the 5G communication strategies [128,129,130,131,132,133,134,135] with enhanced key performance indicators (KPIs) and a wider range of real-world applications. Both 5G and 6G technologies can offer substantially greater computing powers to further improve an AI system’s abilities to generate empirical-based intelligence [128,129,130,131,132,133,134,135]. Research shows that, for instance, analyzing social media posts can offer a grounded and timely insight into citizens’ needs and wants, as well as concerns and considerations in times of crisis such as the COVID pandemic [136,137,138]. Emerging insights also suggest that even small local governments in the U.S. have integrated social media platforms, such as Facebook and Twitter, into their government functions [139], aiming to proactively incorporate public participation in the policy-making process.

### 5.3. Data-Driven

Data-driven entails the need to anchor key policy-making decisions upon the support of empirical evidence abstracted from quality data of great quantity and diversity [99,100,101,102]. Data-driven can refer to either big data analytics or data analyses based on smaller-scale databases. The importance of the data-driven element in the PADS model centers on the use and application of empirically gained insights, as opposed to subjective ideas, in the policy-making process. It is important to underscore that, thanks to advanced technologies such as 5G/6G and AI, a bevy of multifaceted information about public needs and preferences can be cost-effectively monitored, ranging from search queries, social media posts, and sewage data to medical records [83]. Having a diverse pool of heterogenous data paired with advanced computing powers provided by 5G/6G technologies and competent analytical skills enabled by AI means that government and health officials can gain a more complete and comprehensive understanding of the public’s perspective and sentiments towards key policy issues.

Data are, essentially, information about people. Depending on how the data were collected, they could either shed light on information on the people from a third-person perspective (e.g., surveillance footage), relevant information provided by the people via the lens of first-person perspective (e.g., digital diary), or information that is less reflective of differences in perspectives or transitory changes (e.g., biomedical data) [140]. In other words, the data-driven strategy could ensure that both the policy-making process and the resultant policies are founded on and reflective of the collective willpower from diverse perspectives. Overall, incorporating empirical evidence in the design, development, and delivery of policies to ensure the specific rules and regulations are in line with the general public’s needs and wants can be understood as a novel approach to public participation in policy making. Public participation can be understood as the involvement of the public in the government’s agenda-setting and decision-making processes [141]. Essentially, by incorporating big data about people, and oftentimes from people, the data-driven policy-making process constitutes a novel way of ensuring that the individual circumstances are sufficiently heard, considered, and reflected in the public policies, without demanding people’s physical presence in the policy-making process.

### 5.4. Supervision-Enhanced

To err is but human, and artificial intelligence is but a human creation. Noticeably, AI is intrinsically flawed in terms of its lack of ability to initiate ethical considerations and moral judgments [142,143]. In other words, regardless of how remarkable the AI-powered data analytical system might become, in light of the inherent flaws of AI systems—intelligent but without consciousness (e.g., ethical and moral considerations) [144,145,146]—it is essential to safeguard AI systems with instrumental human involvement, in the forms of both policy making by government and health officers and rigorous supervision by independent experts [106]. In other words, to effectively prevent AI from “augmenting disparities” [103] and fostering its abilities to address inequalities or accelerate integrity, sufficient supervision is needed.

Supervision can be understood as oversight mechanisms that scrutinize the behaviors of both the policy makers and the AI systems to further enhance the policies’ abilities to produce positive outcomes without incurring unintended consequences [69,103,104,105,106,107,108]. By rigorously leveraging the supervision-enhanced strategy, the PADS model could help society at large better limit or eliminate potential unintended consequences that could emerge in the policy development, deployment, or delivery processes. One way to form the supervision system is via incorporating an independent review board with rigorously vetted experts participating in the review board on a rotating basis. Other approaches, such as global collaboration [60,147], potentially paired with expertise from international health organizations such as the World Health Organization, may also work. Overall, in light of the multifaceted nature of the concept of “unintended consequences”, it is important to note that, while the presence and robustness of the supervision system are of utmost significance, having an “expert-review-needed” or supervision-needed mindset among policy makers is of equal importance.

One way to view unintended consequences is that they could either be a result of unplanned or unforeseen policy planning—”unplanned” refers to situations in which the negative outcomes are unintended but nonetheless not unanticipated [148], whereas “unforeseen” refers to scenarios in which policy makers were completely unaware of the potential unintended consequences. In other words, not all unintended consequences denote innocence and ignorance on the part of policy makers’—some policies might be made as a result of balancing pros and cons, which means that the welfare of some members of the society could be arbitrarily ignored or neglected during the policy-making process. These flaws could be reflected in AI systems as well [103], which could further compound the potential unintended consequences caused by the policies. A “supervision-needed” mindset could be the solution:

(1) it could facilitate the establishment of policy-making practices that value the importance of supervision;
(2) it could help policy makers avoid causing “unforeseen” consequences in the policy-making process;
(3) it could help policy makers incorporate moral and ethical considerations, ranging from fairness, equality, and privacy to security concerns, in the policy-making process.

### 5.5. The Advantages of the PADS Model

**Figure 1:**
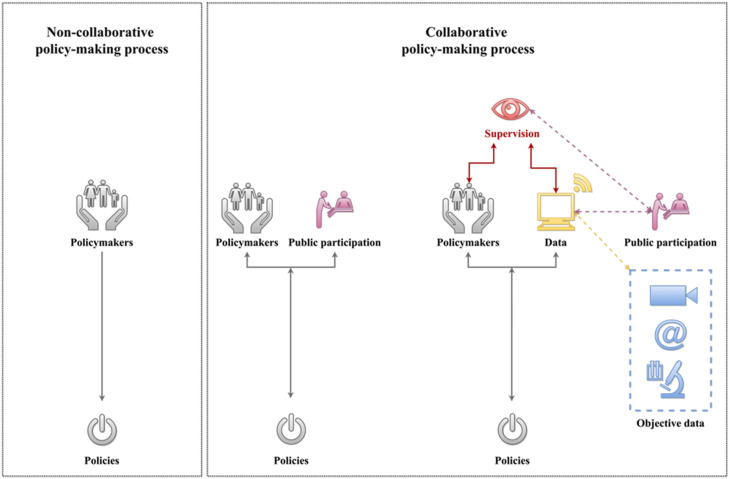
A schematic representation of noncollaborative and collaborative policy-making processes.

In line with the principle of parsimony [149], the policy-making process could be simplified into two collaborative and non-collaborative processes [60,150]. A non-collaborative policy-making process often only involves policy makers. In other words, stakeholders’ input or feedback is often not involved in the process. On the other hand, the collaborative policy-making process not only involves the policy makers, but also stakeholders as well. As the process of policy making evolves, the degree of stakeholder involvement differs across contexts. However, regardless of how the collaboration takes place, this collaborative policy-making process nonetheless suffers from a key flaw—oftentimes both the policymakers and the stakeholders’ input are subjective. A schematic representation of these two policy-making approaches can be found in Figure 1.

Essentially, the leap from noncollaborative policy-making processes to collaborative policy-making processes only addresses one issue in the practice—the lack of public involvement in the decision-making process. In other words, though policies produced via the collaborative policy-making process might have greater abilities to address people’s needs and wants, they nonetheless could be flawed due to the highly subjective nature of the data upon which they are developed. One way to further improve the collaborative policy-making process is via replacing highly subjective and cross-sectional physical public participation with accumulated data that capture both the subjective and the objective needs and preferences of the stakeholders. In other words, data from the stakeholders (e.g., surveys), combined with data about the stakeholders (e.g., third-person perspective data such as surveillance footage, internet activities, etc.) and data about the overall situation from a multitude of perspectives, could serve as a considerably improved virtual proxy of public participation.

As evidence suggests, the general public may be well justified regarding whether or to what degree they wish to comply with COVID-19 public policies [112,113,114,115,116,117]. It is also worth noting that many, if not all, of the COVID-19 public policies were developed based on a top-down approach [151,152], and often without following the proper procedures that allow public participation in the policy-making process [153,154]. Though oftentimes public policy is held as a belief by some governments that “the governments decide to do or not to do” [155], as seen from COVID-19, for the greater good (e.g., achieve a post-pandemic reality), it should be considered and treated as a people-centered ecosystem that aims to serve the general public needs and preferences.

In other words, the data-driven component of PADS can effectively address issues that have been long plaguing policy-making: cross-sectional surveys about people’s needs and preferences are often flawed in offering stable and definitive insights about people, and longitudinal studies are often resource-dependent to conduct or limited in their abilities to provide timely insights into the subject matter. These insights combined suggest that the data-driven characteristics of the PADS model also share advantages that are commonly seen in general public participation in policy-making. It could:

(1) better capture and comprehend the public’s needs and preferences;
(2) design and develop public policies that are grounded in reality and people-centric; and in turn;
(3) yield more desirable policy outcomes and limit potential unintended consequences [141,156,157,158,159].

**Figure 2:**
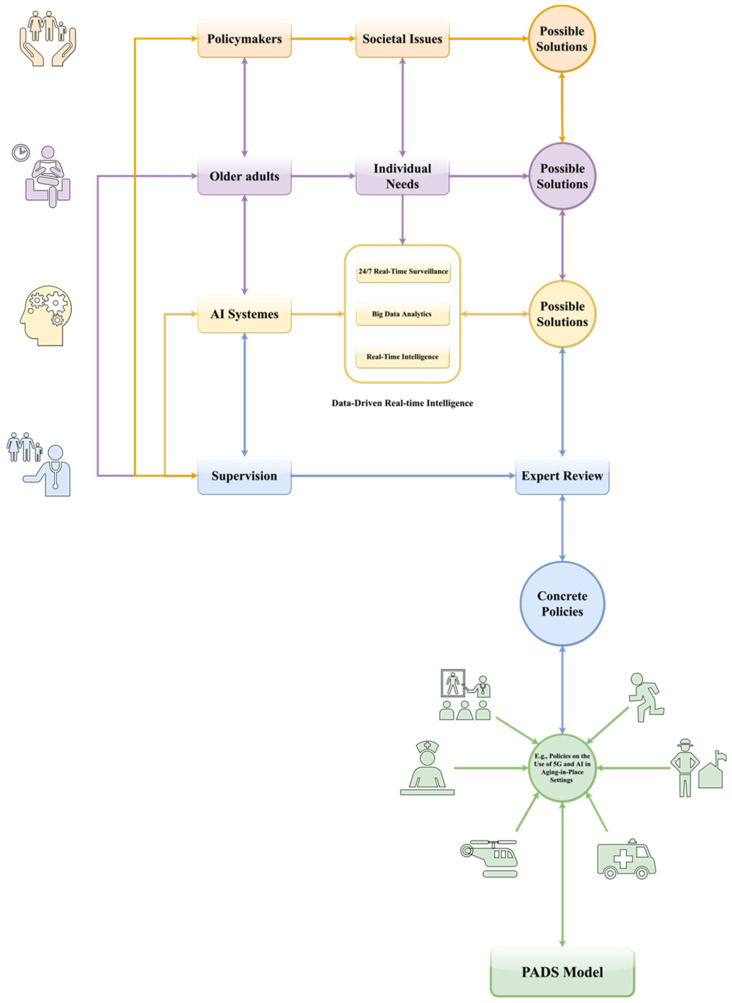
An example utilization of the PADS model in the context of aging-in-place policies.

An example of applying the PADS model for developing policies on the use and application of 5G and AI technologies in the context of aging-in-place can be found in Figure 2. Overall, Figure 2 illustrates how the people-centeredness of the PADS model respects and reflects key stakeholders’ needs and preferences in the policy-making process, with the aid of advanced technologies such as AI-powered systems and comprehensive supervision mechanisms.

### 5.6. Limitations

While this study bridged important research gaps, it was not without limitations. For starters, the review only focused on relevant articles published in the context of the COVID-19 pandemic. This means that potentially valuable insights that were not COVID-19-specific were not included in the review. Due to the focus of the study, challenges such as developmental hurdles associated with the use and application of AI were not discussed in detail in the study. Furthermore, due to the conceptual nature of the PADS model, no empirical evidence about its real-world efficacy is available at the moment. While the efficacies of public health policies could be difficult to evaluate [46], future studies could nonetheless explore innovative approaches to gauge the effectiveness of the PADS model in generating promising policies.

## 6. Conclusions

Policies can be the defining factor in shaping personal and public health, especially amid global catastrophes such as COVID-19. Amid the ever-increasingly chaotic jungle of COVID-19 policy making and the rapidly intensifying public expectations of greater accountabilities among policymakers, it is then vital to investigate rigorous policy-making strategies that could help societies at large develop more cost-effective COVID-19 policies. Based on insights gained from reviewing and analyzing the state-of-the-art evidence in the literature, this study developed the PADS model, which proposes a people-centered, AI-powered, data-driven, and supervision-enhanced approach towards policy making amid COVID-19. The PADS model can develop policies that have the potential to induce optimal outcomes and limit or eliminate unintended consequences amid COVID-19 and beyond. Rather than serving as a definitive answer to problematic COVID-19 policy-making practices, the PADS model can be best understood as one of many promising frameworks that could bring the pandemic policy-making process more in line with the interests of societies at large; in other words, more cost-effectively, and consistently anti-COVID and pro-human.

## Figures and Tables

**Table 1:** Example PubMed search strings.

Concept	Search Strings
Policy making	“policy making” [MeSH] OR “policy making” [TIAB] OR “policy-making” [MeSH] OR “policy-making” [TIAB] OR “policy” [MeSH] OR “policy” [TIAB] OR “policies” [TIAB]
COVID-19	((coronavirus OR “corona virus” OR coronavirinae OR coronaviridae OR betacoronavirus OR covid19 OR “covid 19” OR nCoV OR “CoV 2” OR CoV2 OR sarscov2 OR 2019nCoV OR “novel CoV” OR “wuhan virus”) OR ((wuhan OR hubei OR huanan) AND (“severe acute respiratory” OR pneumonia) AND (outbreak)) OR “Coronavirus” [Mesh] OR “Coronavirus Infections” [Mesh] OR “COVID-19” [Supplementary Concept] OR “severe acute respiratory syndrome coronavirus 2” [Supplementary Concept] OR “Betacoronavirus” [Mesh])

**Table 2:** List of articles included in the final review.

Author	Year	Title
Adiga et al. [65]	2020	Data-driven modeling for different stages of pandemic response
Amanda et al. [66]	2021	Leveraging administrative data for bias audits: Assessing disparate coverage with mobility data for COVID-19 policy
Baker et al. [67]	2020	Elimination could be the optimal response strategy for covid-19 and other emerging pandemic diseases
Baruner Jan et al. [68]	2021	Inferring the effectiveness of government interventions against COVID-19
Bertozzi et al. [63]	2020	The challenges of modeling and forecasting the spread of COVID-19
Blasimme et al. [69]	2020	What’s next for COVID-19 apps? Governance and oversight
Brooks-Pollock et al. [70]	2021	Modelling that shaped the early COVID-19 pandemic response in the UK
Christensen et al. [71]	2020	Balancing governance capacity and legitimacy: How the Norwegian government handled the COVID-19 crisis as a high performer
Duffey et al. [72]	2020	COVID-19 pandemic trend modeling and analysis to support resilience decision-making
Frauke et al. [73]	2020	Partnering with a global platform to inform research and public policy making
Harrison et al. [74]	2020	Data, politics and public health: COVID-19 data-driven decision making in public discourse
Hasan et al. [75]	2021	Data-driven modeling and forecasting of COVID-19 outbreak for public policy making
Lee et al. [76]	2020	Policy learning and crisis policy-making: quadruple-loop learning and COVID-19 responses in South Korea
Liu et al. [77]	2020	Striking a balance between science and politics: understanding the risk-based policy-making process during the outbreak of COVID-19 epidemic in China
Manski [78]	2020	Forming COVID-19 policy under uncertainty
Maor et al. [62]	2020	Explaining variations in state COVID-19 responses: psychological, institutional, and strategic factors in governance and public policy-making
Mazey et al. [47]	2020	Lesson-drawing from New Zealand and COVID-19: the need for anticipatory policy making
Ning et al. [79]	2020	China’s model to combat the COVID-19 epidemic: a public health emergency governance approach
Panovska-Griffiths et al. [80]	2021	Mathematical modeling as a tool for policy decision making: Applications to the COVID-19 pandemic
Qiu et al. [81]	2021	Data-driven modeling to facilitate policymaking in fighting to contain the COVID-19 pandemic
Sartor et al. [82]	2020	COVID-19 in Italy: Considerations on official data
Su et al. [83]	2020	Addressing Biodisaster X threats with artificial intelligence and 6G technologies: Literature review and critical insights
Totsoy [61]	2021	COVID-19 epidemic and opening of the schools: artificial intelligence-based long-term adaptive policy making to control the pandemic diseases
Ullah et al. [58]	2021	The role of e-governance in combating COVID-19 and promoting sustainable development: A comparative study of China and Pakistan
Willi et al. [84]	2020	Responding to the COVID-19 crisis: Transformative governance in Switzerland
Yu et al. [85]	2021	Data-driven decision-making in COVID-19 response: A survey
Zawadzki et al. [86]	2021	Where do we go from here? A framework for using susceptible-infectious-recovered models for policy making in emerging infectious diseases
Zheng et al. [87]	2020	HIT-COVID, a global database tracking public health interventions to COVID-19

## Abbreviations

COVID-19	Coronavirus disease 2019
U.S.	United States
